# Beyond Gleason grading: MRI radiomics to differentiate cribriform growth from non-cribriform growth in prostate cancer men

**DOI:** 10.1007/s10334-025-01251-5

**Published:** 2025-04-29

**Authors:** Mar Fernandez Salamanca, Rita Simões, Malgorzata Deręgowska-Cylke, Pim J. van Leeuwen, Henk G. van der Poel, Elise Bekers, Marcos A. S. Guimaraes, Uulke A. van der Heide, Ivo G. Schoots

**Affiliations:** 1 Department of Radiology, The Netherlands Cancer Institute, Plesmanlaan 121, 1066CX Amsterdam, The Netherlands; 2 Department of Radiation Oncology, The Netherlands Cancer Institute, Amsterdam, The Netherlands; 3 Department of Radiology, Medical University of Warsaw, Warsaw, Poland; 4 Department of Urology, The Netherlands Cancer Institute, Amsterdam, The Netherlands; 5 Department of Urology, Amsterdam University Medical Centers, Amsterdam, The Netherlands; 6 Department of Pathology, The Netherlands Cancer Institute, Amsterdam, The Netherlands; 7 Department of Radiology and Nuclear Medicine, Erasmus University Medical Center, Rotterdam, The Netherlands

**Keywords:** MRI, Radiomics, ADC, Prostatectomy, Cribriform growth

## Abstract

**Objective:**

To differentiate cribriform (GP4Crib+) from non-cribriform growth and Gleason 3 patterns (GP4Crib-/GP3) using MRI.

**Methods:**

Two hundred and ninety-one operated prostate cancer men with pre-treatment MRI and whole-mount prostate histology were retrospectively included. T2-weighted, apparent diffusion coefficient (ADC) and fractional blood volume maps from 1.5/3T MRI systems were used. 592 histological GP3, GP4Crib- and GP4Crib+ regions were segmented on whole-mount specimens and manually co-registered to MRI sequences/maps. Radiomics features were extracted, and an erosion process was applied to minimize the impact of delineation uncertainties. A logistic regression model was developed to differentiate GP4Crib+ from GP3/GP4Crib- in the 465 remaining regions. The differences in balanced accuracy between the model and baseline (where all regions are labeled as GP3/GP4Crib-) and 95% confidence intervals (CI) for all metrics were assessed using bootstrapping.

**Results:**

The logistic regression model, using the 90th percentile ADC feature with a negative coefficient, showed a balanced accuracy of 0.65 (95% CI: 0.48–0.79), receiver operating characteristic area under the curve (AUC) of 0.75 (95% CI: 0.54–0.92), a precision–recall AUC of 0.35 (95% CI: 0.14–0.68).

**Conclusion:**

The radiomics MRI-based model, trained on Gleason sub-patterns segmented on whole-mount specimen, was able to differentiate GP4Crib+ from GP3/GP4Crib- patterns with moderate accuracy. The most dominant feature was the 90th percentile ADC*.* This exploratory study highlights 90th percentile ADC as a potential biomarker for cribriform growth differentiation, providing insights into future MRI-based risk assessment strategies.

## Introduction

Magnetic resonance imaging (MRI) has become an indispensable tool in the diagnosis and management of prostate cancer. Beyond its ability to exclude significant disease and reduce the need for unnecessary biopsies, MRI provides detailed insights into cancer-suspected tissues, guiding precise lesional targeting and improving tissue characterization. Consequently, MRI is now recommended for individualized prostate cancer risk assessment in men with an increased risk and previously considered for biopsy [1–6].

Within this risk assessment framework, the histological subtype of cribriform growth, a Gleason 4 sub-pattern, has gained attention due to its association with worse clinical outcomes when present [7, 8]. Histologically proven presence of cribriform growth is correlated with lower apparent diffusion coefficient (ADC) values in MRI-suspected lesions [9, 10]. This association is particularly pronounced in high-risk (high Gleason score) men, where cribriform growth is more prevalent, indicating a more aggressive disease phenotype [10].

In contrast to high-risk men, intermediate-risk men present a diverse spectrum of clinical outcomes, which are significantly influenced by the presence of specific Gleason 4 subtypes, especially cribriform growth [11, 12]. While treatment is universally recommended for high-risk men, some intermediate-risk men may be candidates for surveillance, provided that cribriform growth can be confidently excluded [6]. For making personalized treatment decisions in this intermediate-risk group, differentiating cribriform from non-cribriform presence is, therefore, of critical importance.

Differentiating cribriform from non-cribriform growth presence is even more challenging in men with International Society of Urological Pathology (ISUP) grade group 2 prostate cancer, where the percentage of Gleason 4 is less than 50% of the whole lesion volume. In these men, the presence of cribriform growth on biopsy is difficult to detect by histopathology analysis [13]. Ideally, imaging biomarkers based on quantitative MRI could support biopsy results interpretation.

This study focuses on the identification of imaging features distinguishing cribriform growth regions from other Gleason pattern 4 and 3 patterns regions, in biopsy-confirmed prostate cancer patients who subsequently underwent robot-assisted radical prostatectomy. The model is intended to be an explanatory analysis, not to be a direct clinical application. We performed an in-depth analysis of histopathological regions on whole-mount specimens, focusing on discriminating cribriform growth from other Gleason Pattern 4 (GP4) subtypes, alongside Gleason Pattern 3 (GP3). To achieve this, we developed an image-based logistic regression model using MRI radiomics features.

## Materials and methods

This study was approved by the institutional review board (IRBd21-108), and informed consent was waived because of the retrospective nature of the study.

**Fig. 1:**
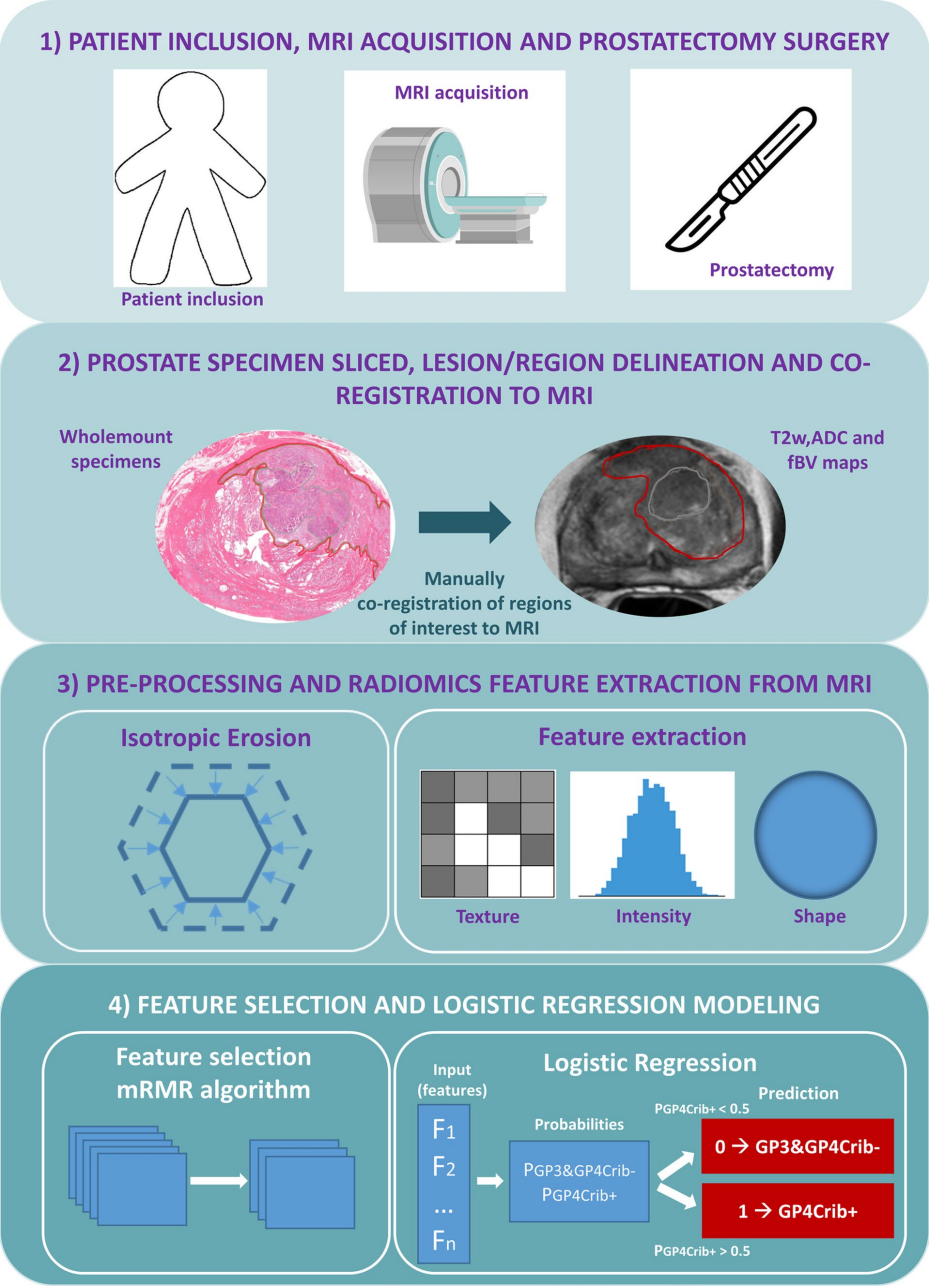
Pipeline used for classification of histopathological regions of cribriform growth based on MRI radiomics. GP3, GP4Crib- and GP4Crib + regions were delineated in whole-mount specimens and subsequently co-registered to MRI. Isotropic erosion was performed in all regions for further radiomics extraction on T2w, ADC and fBV scans. For model development, mRMR selection algorithm was used and selected features were used as input for the logistic regression model to predict GP4Crib+ . Model outcome was shown as a predicted probability of the positive class (GP4Crib+) and binary label (0: GP3 and GP4Crib-; 1: GP4Crib+). T2w: T2-weighted images; ADC: apparent diffusion coefficient; fBV: fractional blood volume; mRMR: minimum redundancy maximum relevance; GP3: Gleason Pattern 3; GP4Crib-: Gleason Pattern 4 subtypes excluding cribriform growth; GP4Crib+ : Gleason Pattern 4 subtype cribriform growth

Men with biopsy-proven prostate cancer and a pre-operative MRI, who underwent radical prostatectomy at our institute between January 2010 and December 2020, were included. Exclusion criteria were men with prior transurethral resection of the prostate or neoadjuvant systemic therapy or radiotherapy, incomplete or technically poor-quality of MRI and pathological specimens. 291 patients were subjected to further analysis (Table 1). The processing pipeline is shown in Fig. 1.

**Table 1:** Patient demographics and characteristics of histopathological segmentations

Finding	Cohort (pre-erosion)	Cohort (post-erosion)
Patients	n = 291	n = 270
Age (y)*	67 (46–78)	66 (46–78)
PSA (ng/ml)*	8.3 (2.5–259)	8.2 (2.5–259)
Radical prostatectomy ISUP gg		
1	33 (12%)	32 (12%)
2	134 (46%)	126 (47%)
3	74 (25%)	72 (26%)
4	29 (10%)	27 (10%)
5	21 (7%)	13 (5%)
Radical prostatectomy T stage		
2	168 (58%)	160 (59%)
3	123 (42%)	110 (41%)
Regions of cancer on histopathology	n = 592	n = 465
GP3	264 (45%)	242 (52%)
GP4Crib-	238 (40%)	176 (38%)
GP4Crib+	90 (15%)	47 (10%)

PSA: Prostate-specific antigen; ISUP gg: International Society of Urological Pathology grade group; GP3: Gleason Pattern 3; GP4Crib-: Gleason Pattern 4 subtypes excluding cribriform; GP4Crib+ : Gleason Pattern 4 subtype cribriform growth

^*^Data are medians (min – max)

### Pre-treatment MRI acquisition protocol and data analysis

MRI scans were acquired at 1.5T (Achieva [n = 3]) and 3T scanners (Achieva [n = 161], Achieva dStream [n = 103] and Ingenia [n = 21], Philips Healthcare, Best, the Netherlands). Axial T2-weighted (T2w) turbo spin-echo images were acquired (repetition time = [2175–10,233 ms], echo time = [110–130 ms]) with a field of view (FOV) from 140 × 140 mm to 284 × 284 mm and slice thickness of 2.5 to 4 mm). 2D single-shot echo planar diffusion-weighted images were acquired with a FOV ranging from 180 × 180 mm to 381 × 381 mm, slice thickness of 2.73 to 4 mm and in plane resolution of 0.56 × 0.56 to 1.2 × 1.2 mm^2^. The group of b-values present in this cohort were 0, 200, and 800 or 0, 50, 300, and 800 s/mm^2^. Full details of the T2w and DWI scanning parameters were published previously [14]. The ADC and fractional blood volume (fBV) maps were calculated using an intravoxel incoherent motion model (IVIM) with a segmented fit approach^1^ [14, 15]. ADC values were calculated using a b-value selection of 200–800 s/mm^2^. Negative ADC and fBV voxel values were set to zero as they lacked physical significance. Rigid registration was performed to align DWI to T2w images, using the prostate mask as a reference.

### Radical prostatectomy specimen processing

Radical prostatectomy specimens were formalin-fixed, transversely sectioned, and entirely embedded. Whole-mount 3 mm slices were used. Histological evaluation (Gleason grading) was performed by a uro-pathologist (Marcos.A.S.Guimaraes., 10 years’ experience) using SlideScore software [https://www.slidescore.com/] using a × 82 magnification.

### Segmentations on whole-mount radical prostatectomy specimen

Lesions larger than 3 mm were graded according to the 2019 International Society of Urological Pathology (ISUP) recommendations [16]. In every cancer lesion, the presence of GP3 and GP4 was segmented separately, more specifically discriminating between GP4 sub-pattern cribriform growth (GP4Crib+) from the other GP4 subtypes (GP4Crib-).

### Co-registration of histopathology segmentations to MRI

Histopathology segmentations (i.e., ground truth) were manually co-registered to T2w MRI by visually aligning histological delineations to MRI images using anatomical landmarks, such as prostate contours, benign hyperplasia nodules, and the urethra. This alignment was achieved by reviewing the histopathology and MRI images simultaneously and matching corresponding structures between the two modalities. While some discrepancies due to tissue deformation during histological processing are inevitable, this manual, anatomy-guided approach represented the most feasible method for co-registration in this study. All MRI segmentations were performed using Slicer version 4.11.20210226 (3D Slicer; https://www.slicer.org).

### Erosion of the MRI regions of co-registered histopathology segmentations

Two primary sources of segmentation uncertainty were identified in the analysis of the MRI regions: (1) the manual co-registration from histopathology to T2-weighted MRI and (2) the partial volume effect, which is particularly significant in smaller volumes. To mitigate these uncertainties, an erosion process was applied to all segmented MRI regions (ndimage.binary_erosion function, SciPy library (version 1.11.1)), effectively removing voxels at the MRI region’s boundary. Isotropic erosion was utilized in a cylindrical structuring element in the XY plane of the ADC map as it is more affected by the partial volume effect. Erosion was applied exclusively to slices with adjacent upper and lower slices, excluding edges prone to delineation uncertainties. The erosion radius was determined such that one acquisition voxel was isotopically removed from the structure. This approach ensures that the extracted features of the regions within the tumor are more representative of the actual underlying Gleason patterns, minimizing contamination from healthy tissue or other patterns at the region boundaries. This approach ensured that the erosion process was tailored to the imaging characteristics of each individual. The erosion process effectively mitigated the impact of registration uncertainties, ensuring more precise alignment and improving the accuracy of subsequent analyses [17].

### Radiomics feature extraction of processed MRI regions

Z-score normalization was used to normalize T2-weighted (T2w) image intensities using the normalize function from Pyradiomics, ensuring consistency across scans. The images were discretized using an optimal bin size of 3, determined to ensure that most scans would result in approximately 10 to 100 bins. To establish this bin size, the first-order (FO) range was calculated across the training and validation sets, and the optimal bin width was determined based on the minimum and maximum range. In this study, the T2w FO range spanned from [6.81–330.4] turned to [2.27–110.12] bins after applying a bin width of 3. Resampling was applied to all T2w, ADC, and fBV scans, with the resampling pixel spacing calculated based on the median voxel size in the training and validation sets combined. When the segmentation mask and input image did not share the same grid, the mask was resampled, within the Pyradiomics pipeline, to the image grid using Nearest-Neighbor interpolation. Details of the settings used for radiomics extraction are provided in Table 2. Only shape and first-order features were extracted from ADC and fBV maps due to their lower spatial resolution. Nevertheless, texture features were extracted from T2w images which provide higher spatial resolution suitable for characterizing voxel intensity distributions. A total of 7 shape features, 19 first-order features and 81 texture features were extracted from their respective maps. The feature extraction was performed using the PyRadiomics package (version 3.1.0) in Python (version 3.8).

**Table 2:** Radiomic extraction settings

Setting	T2w	ADC & fBV
Normalize	True	False (default)
Normalize scale	100	1 (default)
Bin width	3	25 (default)
Interpolator	sitkBSpline (default)	sitkBSpline (default)
Resampled pixel spacing (mm)	[0.27, 0.27, 3]	[1.03, 1.03, 2.73]
Minimum ROI dimension	1	1
Correct mask	True	False (default)
Geometry tolerance	1e-4	1e-16 (default)
Extracted features	glcm, glrlm, glszm, ngtdm, gldm	Shape and first order

All segmentation masks were in the same grid and size as the ADC and fBV maps. T2w: T2 weighted; ADC: Apparent diffusion coefficient; fBV: fractional blood volume. Other settings were used as default and can be found in the Pyradiomics documentation; https://pyradiomics.readthedocs.io/en/latest/customization.html#radiomics-parameter-file-label

### Radiomics model development and validation

The radiomics model was designed to predict GP4Crib+ regions, while prioritizing the optimization of the balanced accuracy. This metric is the arithmetic mean of sensitivity and specificity and is used when dealing with imbalanced data. Processed MRI regions were randomly split into training (60%), validation (20%), and test (20%) sets, ensuring a balanced distribution of classes and volume among the sets (Table 3). A radiomics (logistic regression—LogReg) model [18] was trained to differentiate GP4Crib+ from GP3 and GP4Crib-. Class weighting was employed to mitigate overfitting and account for class imbalance, respectively. Other parameters were further optimized (Table 4). Feature selection was performed using the mRMR algorithm [19], which ranked features based on their mutual information with the target variable (GP4Crib+) while minimizing redundancy with other features. The output features from the mRMR algorithm were iteratively added to the model one at a time, with a maximum of 20 features allowed to ensure model interpretability. Features were not explicitly excluded based on their interpretability, as the algorithm ranked them strictly by statistical relevance and redundancy. The optimal number of features, as well as the parameter values, were determined in the validation set using threefold cross-validation, determined by the set of features giving the highest balanced accuracy value. Sensitivity and specificity values were extracted with the addition of receiving operating characteristic area under the curve (ROC AUC) and precision–recall AUC values. Predicted probabilities of cribriform growth regions were calculated using the logistic regression formula:

$$P = {1 \mathord{\left/ {\vphantom {1 {1 + e^{{ - \left( {w_{0} + w_{1} \times X_{1} + w_{2} \times X_{2} + \cdots + w_{n} \times X_{n} } \right)}} }}} \right. \kern-0pt} {1 + e^{{ - \left( {w_{0} + w_{1} \times X_{1} + w_{2} \times X_{2} + \cdots + w_{n} \times X_{n} } \right)}} }}$$

where ${X}_{i}\equiv$ *features* of a given region and ${w}_{i}$ are the coefficients of logistic regression. A binary outcome was then achieved by applying a 0.5 threshold to these probabilities.

**Table 3:** Training, validation and test sets of eroded MRI regions of cribriform and non-cribriform histological growth patterns, segmented on whole-mount radical prostatectomy specimen

Sets	GP3	GP4Crib-	GP4Crib+
All (n = 465)	242 (52)	176 (38)	47 (10)
V (cc)*	0.16 (0.006–28.94)	0.20 (0.002–12.00)	0.08 (0.006–3.01)
Training (n = 279)	145 (52)	106 (39)	28 (10)
V (cc)*	0.16 (0.006–28.94)	0.20 (0.002–12.00)	0.08 (0.009–1.29)
Validation (n = 93)	49 (52)	35 (38)	9 (10)
V (cc)*	0.16 (0.006–2.90)	0.20 (0.007–5.94)	0.08 (0.006–3.01)
Test (n = 93)	48 (52)	35 (38)	10 (10)
V (cc)*	0.14 ( 0.006–2.81)	0.21 (0.009–3.47)	0.08 ( 0.01–0.97)

Unless otherwise specified, data are numbers with percentages in parenthesis. GP3: Gleason Pattern 3; GP4Crib-: Gleason Pattern 4 subtypes excluding cribriform; GP4Crib+ : Gleason Pattern 4 subtype cribriform growth; V: Volume in cc

*Data are medians (min–max)

**Table 4:** Logistic regression parameters used for model optimization and optimal model

Parameter	Values	Final model
C	[0.001, 0.01, 0.1, 1, 2, 10]	1
penalty	[‘l1’, ‘l2’, ‘elasticnet’]	‘l2’
Max_iter	[10,50,100,500]	10
Class_weight	[‘balanced’, (0: 0.2, 1: 0.8), (0:1, 1:10)]	‘balanced’
Fit_intercept	[True, False]	True

All parameters are explained in Scikit learn; https://scikit-learn.org/LogisticRegression

### Statistical analysis

The difference in balanced accuracy values between the model and a baseline (where all regions are labeled as GP3/GP4Crib- representing a balanced accuracy of 0.5) and 95% confidence intervals (CI) for all metrics were assessed using bootstrapping with n = 1000. All analyses were conducted using Python v3.11.

## Results

Within this cohort of 291 operated prostate cancer men, 592 regions of GP3 (n = 264), GP4Crib- (n = 238) and GP4Crib+ (n = 90) were segmented on histology whole-mount radical prostatectomy specimens and registered to T2w scans (Table 1). Following the erosion process, 107 regions were removed from the 592 regions, because no voxels remained after erosion. From the remaining 485 eroded regions, another 20 regions were discarded as radiomics extraction was not possible (only one voxel was left in the segmentation). Radiomics features were extracted from the remaining 465 MRI regions (Table 1), with resampled voxel size and bin size settings as detailed in Table 2, ensuring consistency across patients for the feature extraction process. The MRI regions were split in the training, validation and test sets, maintaining both the histological proportion and the volume distribution (Table 3). A control analysis was conducted using the 592 non-eroded regions to assess potential biases introduced by the erosion process. This analysis revealed that median ADC and zone entropy T2w were the features driving the classification in non-eroded regions, both with negative coefficients. Zone entropy T2w, which measures the randomness or heterogeneity of texture patterns, became more relevant in the presence of smaller regions prior to erosion (many of which are cribriform and more likely to be affected by delineation uncertainties). In addition, the analysis of non-eroded regions indicated that the low median ADC may reflect contamination by higher ADC values from non-cribriform tissue. In comparison, the model using eroded regions identified a low 90th percentile ADC, which suggests the absence of high ADC values within the volume. These observations complement each other and support the hypothesis that the erosion process reduces noise and mitigates contamination from adjacent tissues and delineation uncertainties, while preserving the information necessary for cribriform differentiation.

### Radiomics model development and validation

**Fig. 2:**
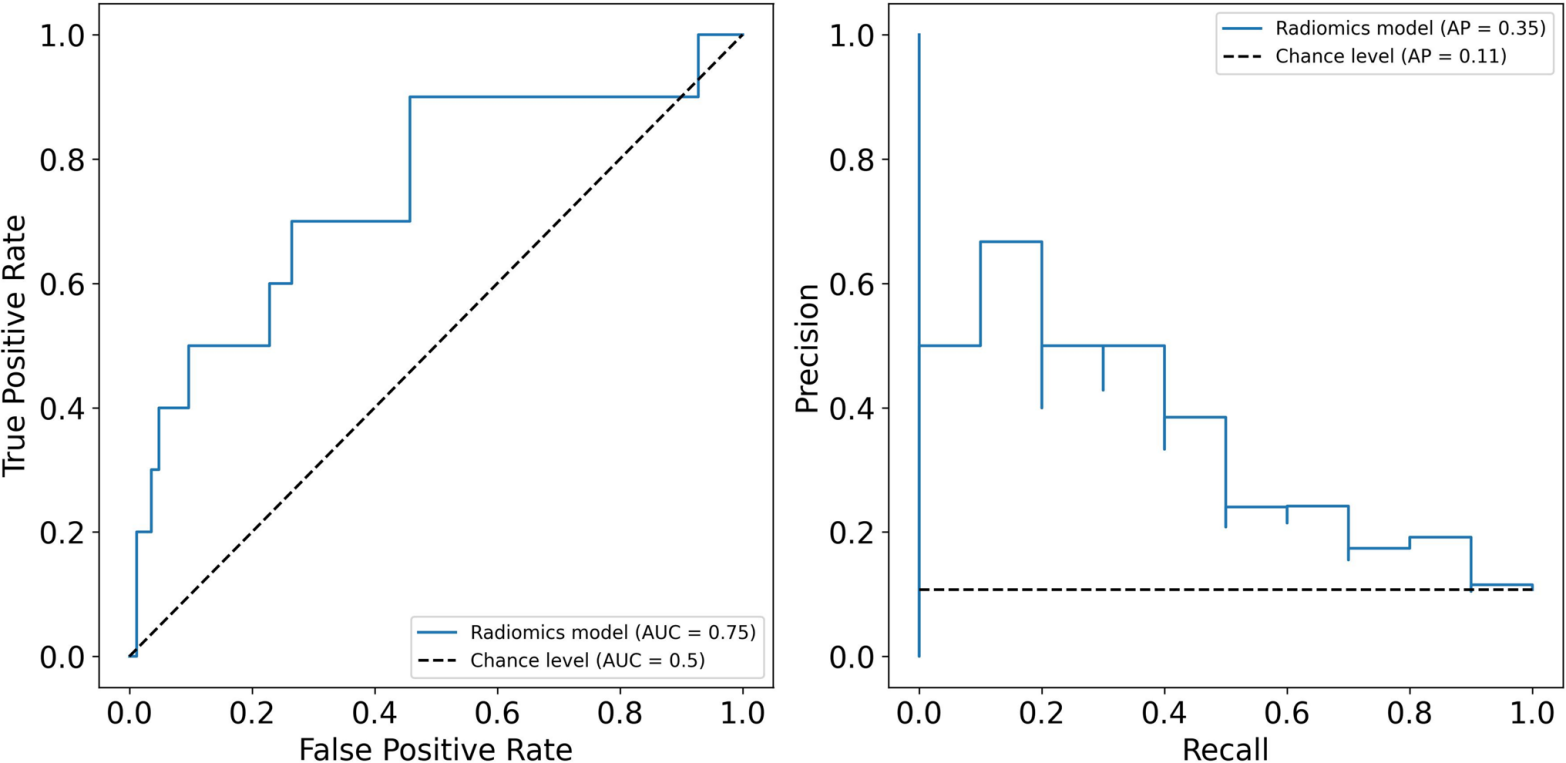
Performance metrics of the radiomics model in the test set. Left plot: ROC curve, right plot: precision–recall curve

The radiomics-based logistic regression model that performed best in the validation set consisted of an optimal L2 regularization parameter value of 1, a class weighting scheme of ‘balanced’ and a maximum number of iterations was 10 (Table 4). Moreover, the model used 90th percentile ADC as the only feature for prediction. In the test set, with a balanced accuracy of 0.65 (95% CI: 0.48–0.79), the model was exceeding the baseline balanced accuracy of 0.50 by an absolute 0.15 (p < 0.05). Finally, the model achieved a ROC AUC of 0.75 (95% CI: 0.54–0.92) and a precision–recall AUC of 0.35 (95% CI: 0.14–0.68), both significantly higher (p < 0.05) than the chance level (Fig. 2). Moreover, a sensitivity and specificity of 0.70 (95% CI: 0.38–1) and 0.59 (95% CI: 0.48–0.69), respectively (Table 5), were obtained for cribriform growth differentiation.

**Table 5:** Radiomics model metrics performance

Metric	Value	95% CI
Balanced accuracy	0.65	0.48–0.79
ROC AUC	0.75	0.54–0.92
PR AUC	0.35	0.14–0.68
Sensitivity	0.70	0.38–1.00
Specificity	0.59	0.48–0.69

ROC AUC: receiving operating characteristic area under the curve, PR AUC: precision–recall area under the curve, CI: confidence interval

The regression analysis revealed an intercept value of w_0_ = 1.96 and a negative coefficient (w_1_ = -1.74) for the 90th percentile ADC feature. This indicates an inverse relationship with the probability of a region being characterized as GP4Crib+ . This means that the higher the 90th percentile ADC value for a given region, the lower the probability of it being characterized as GP4Crib+ . The model found a 90th percentile ADC region value of 1.13×10^−3^ mm/s^2^ corresponding to a predicted probability of 50% for a region being characterized as GP4Crib+ . Thus, regions with 90th percentile ADC < 1.13×10^−3^ mm/s^2^ were labeled as cribriform growth, while regions with 90th percentile ADC > 1.13×10^-3^ mm/s^2^ were labeled as non-cribriform.

### Explainable case-based examples of the model performance

**Fig. 3:**
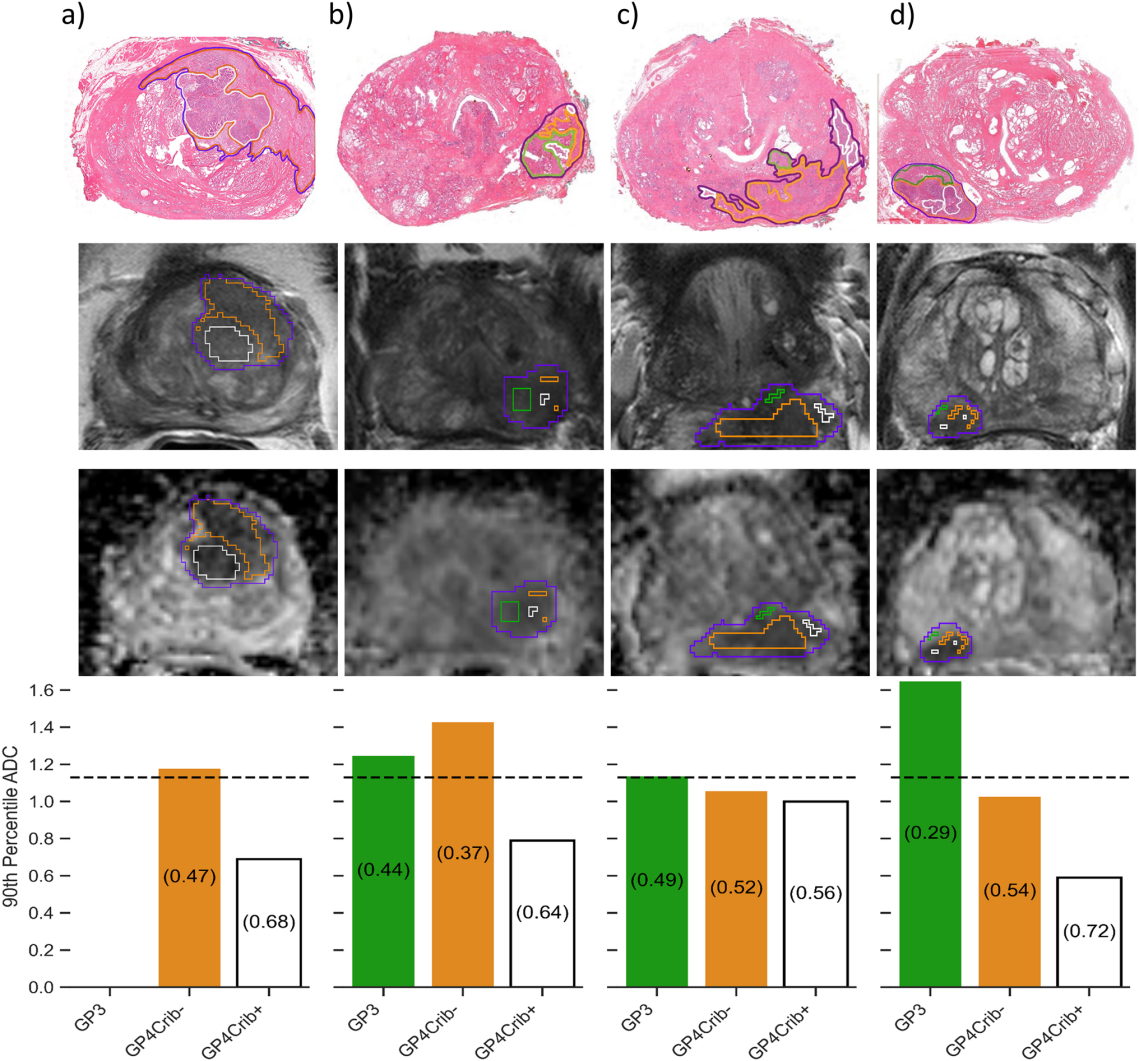
Model outcome of four prostate cancer examples. Lesion (purple), Gleason Pattern 3 (green), Gleason Pattern 4 subtypes excluding cribriform growth (orange) and Gleason Pattern 4 subtype cribriform growth (white)). The horizontal dotted line at the 90th percentile ADC of 1.13×10^−3^ mm/s^2^ corresponds to a predicted probability of 50% chance for any region being characterized as GP4Crib+ . Numbers between brackets show the predicted probability. Taking a cutoff of 1.13×10^−3^ mm/s^2^, regions with the 90th percentile ADC below the cutoff are classified as positive for GP4Crib+ , whereas regions above the cutoff are classified as negative. [A] ISUP Grade Group 4 cancer in the left transition zone shows two regions, GP4Crib- and GP4Crib+ . Both regions were correctly predicted. [B] IUSP Grade Group 2 cancer in the left peripheral zone shows three regions, GP3, GP4Crib- and GP4Crib+ , which were correctly predicted, respectively, being true negative, true negative and true positive. [C] ISUP Grade Group 3 cancer in left peripheral zone shows three regions. While the GP4Crib+ region was correctly predicted (true positive), GP3 and GP4Crib- regions were both falsely predicted as GP4Crib+ positives. Despite the overdiagnosis (false positives), there was no underdiagnosis (no false negatives).The overall presence of cribriform growth within this patient was adequately assessed. [D] ISUP Grade Group 3 cancer in the right peripheral zone shows three regions. For the GP4Crib+ and GP3 regions, the model correctly predicted the presence and absence of cribriform growth, being true positive and true negative, respectively. However, for GP4Crib-, the model incorrectly predicted the presence of cribriform growth, being false positive, contributing to overdiagnosis

Four case-based examples illustrate the model performance on an individual basis, showing the predicted probabilities for GP4Crib+ growth in the prostate cancer segmented regions, in respect to the 90th percentile ADC values (Fig. 3). These examples highlight the complexity of the analyses regarding the differences in zonal location, lesion/tumor volume, regional surface area, and Gleason grading (including GP4Crib+ / GP4Crib- sub-patterns).

## Discussion

In this study, a radiomics-based logistic regression model was developed using prostate MRI images and detailed histological review of radical prostatectomy whole-mount specimens. The model was able to potentially differentiate GP4Crib+ pattern from GP3/GP4Crib- patterns in men who underwent a radical prostatectomy. Cribriform growth is characterized by densely packed cellular structures and reduced extracellular space which decreases the diffusion of water molecules. As a result, cribriform growth regions exhibit lower ADC values compared to other Gleason patterns. This biological basis supports the imaging findings of this study, where lower 90th percentile ADC values were predictive of cribriform growth regions.

Previous studies that have focused on the appearance of tumors with cribriform growth pattern on MRI have shown various results. Truong et al. [20] showed that the size threshold for identifying cribriform growth tumors was significantly higher than for other patterns, making MRI-targeted biopsies less effective than systematic biopsies. Subsequently, Tonttila et al. [21] showed that, in their prostatectomy cohort, multiparametric MRI had a high sensitivity for cancer detection including the cribriform growth cancers. However, reliable prediction of ISUP grade group 2 cancers with cribriform growth remained challenging [21]. These discrepancies between studies regarding the identification and visibility of cribriform growth cancers could be explained by differences in histopathological classification [22]. Tonttila et al. [21] considered cribriform growth disease when combined with other Gleason patterns, resulting in an easier visualization on MRI, while Truong et al. [20] focused on pure cribriform growth tumors. Due to these inconsistencies and the importance of identifying cribriform growth in prostate cancer men, especially in men with potential favorable disease (i.e., ISUP gg 2 disease), we aimed to study the MRI features of histologically proven GP3 and GP4, including cribriform growth regions, separately (Fig. 3).

In this study, the 90th percentile ADC feature emerged as the most predictive feature for excluding GP4Crib+ regions, achieving the highest balanced accuracy and AUC in the validation set after cross-validation. Adding more features did not improve the performance of the model. By focusing on Gleason sub-pattern regions rather than the entire lesions, we reduced the heterogeneity in prostate cancers. This reduction in heterogeneity explains why a single feature emerged as sufficient for differentiation. Although the 90th percentile ADC may seem counterintuitive initially, given that lower ADC values are typically associated with more aggressive cancer phenotypes (such as cribriform growth pattern), the model aligns with this relationship due to its inverse relation with this feature. Specifically, a lower 90th percentile ADC value confirms the presence of GP4Crib+ in a given region, indicating that the ADC values in that area tend to be lower. On the other hand, higher ADC values are associated with a lower likelihood of cribriform growth, meaning that in regions where cribriform growth is absent, higher ADC values are observed. These areas with less restricted diffusion represent tissue cellularity or micro environmental factors linked to less aggressive cancerous growth patterns that contribute to the differentiation of GP4Crib+ from GP3/GP4Crib- regions [17]. Caution is warranted when generalizing these findings to higher ISUP grade group populations, where the prevalence of GP4Crib+ may be higher.

One of the strong points of this study is the detailed histological review of radical prostatectomy whole-mount specimens and further mapped to T2w MRI scans. To the best of our knowledge, this level of detailed histopathological annotation is not common, making this dataset unique. This comprehensive approach lays a foundation for future research in imaging-based modeling for cribriform growth exclusion in prostate cancer. By enhancing our understanding of cancer heterogeneity and its Gleason subtypes, particularly GP4Crib+ , we can improve the accuracy and reliability of prostate cancer diagnosis, prognosis, treatment decision-making, and planning.

Some limitations should be acknowledged. The number of patients is limited and derived from one single center retrospectively, limiting the power analysis. In addition, the predominance of low- and intermediate-grade group patients resulted in a limited prevalence of cribriform growth regions. Due to the lack of published datasets providing detailed Gleason sub-pattern segmentation as required for this study, external validation was not explored. Another significant challenge in this study was the inherent uncertainty in aligning histopathological delineated regions with their corresponding MRI regions during the co-registration process. This misalignment could lead to contamination from nearby tissues (either other Gleason sub-patterns or healthy tissue), introducing errors in feature extraction. This issue is particularly relevant for smaller GP4Crib+ regions, which are more prone to MRI partial volume effects. To mitigate these issues, an erosion process was applied to all regions, reducing the prevalence of GP4Crib+ to 10%, which is relatively low for diagnostic modeling. However, by addressing the challenge of histological heterogeneity within prostate cancer lesions—particularly focusing on cribriform growth pattern—this study provides foundational insights that can inform future research. Finally, ADC values, while associated with cribriform growth, can overlap with other GP4Crib- regions, leading to potential misclassifications. Regions with dense stromal phenotype, irrespective of Gleason pattern, may exhibit low 90th percentile ADC values and be misclassified as GP4Crib+ , as demonstrated in Fig. 3.

This radiomics-based logistic regression model, based on prostate MRI images and detailed histological review of radical prostatectomy whole-mount specimens, was able to differentiate cribriform from non-cribriform growth patterns. The 90th percentile ADC feature was identified as the most predictive feature for this differentiation. This work serves as an exploratory analysis aimed at identifying imaging signatures distinguishing GP4Crib+ regions from GP3 and GP4Crib- regions rather than classifying entire (heterogeneous) tumors as having cribriform growth or not. Although the precision–recall AUC is relatively low, this study supports the potential of ADC as a biomarker for cribriform growth identification and represents a crucial step in addressing the histological heterogeneity of prostate cancer lesions. Excluding cribriform growth is particularly relevant for the growing population of intermediate-risk men, potentially distinguishing favorable from unfavorable disease and guiding personalized treatment decisions in respect to surveillance or radical treatment. For clinical translation, future research should focus on using these imaging signatures to better localize cribriform regions which would be useful information for biopsy targeting or radiotherapy boosting plans and treatment.

## Data Availability

Data that support the findings of this study are not openly available due to reasons of sensitivity.

## Footnotes

1 https://github.com/OSIPI/TF2.4_IVIM-MRI_CodeCollection/tree/main/src/original/PvH_KB_NKI.
